# First detection and molecular characterization of porcine reproductive and respiratory syndrome virus in Namibia, Africa

**DOI:** 10.3389/fvets.2023.1323974

**Published:** 2024-01-15

**Authors:** Umberto Molini, Lauren M. Coetzee, Maria Y. Hemberger, Bernard Chiwome, Siegfried Khaiseb, William G. Dundon, Giovanni Franzo

**Affiliations:** ^1^School of Veterinary Medicine, Faculty of Health Sciences and Veterinary Medicine, University of Namibia, Windhoek, Namibia; ^2^Central Veterinary Laboratory (CVL), Windhoek, Namibia; ^3^Faculty of Veterinary Medicine, University of Teramo, Teramo, Italy; ^4^Animal Production and Health Laboratory, Animal Production and Health Section, Department of Nuclear Sciences and Applications, Joint FAO/IAEA Division, International Atomic Energy Agency, Vienna, Austria; ^5^Department of Animal Medicine, Production and Health, University of Padova, Legnaro, Legnaro, Italy

**Keywords:** PRRSV, Namibia, Africa, molecular epidemiology, phylogenetics, virus

## Abstract

**Introduction:**

The swine sector in Africa plays an important role in local economies, contributing to poverty alleviation and community subsistence. In addition, intensive farming is progressively becoming more important in the region. Therefore, any disease affecting swine populations can have detrimental effects on local communities. Porcine Reproductive and Respiratory Syndrome (PRRS) is among the most important infectious diseases affecting swine worldwide, but information on its epidemiology in Africa is extremely limited.

**Material and methods:**

In the present study, 147 healthy butchered pigs, originating from 15 Namibian intensive and rural farms were tested by RT-PCR and the ORF7 genes of positive samples were sequenced for further genetic characterization and phylogenetic analysis. Additionally, 55 warthogs were also evaluated using the same approach.

**Results:**

Overall, 7 out of 147 pigs (4.76%) tested positive, all originating from 3 rural farms (with a within-herd detection frequency higher than 14%) characterized by strong epidemiological links. All industrial pig and warthog samples were negative. Sequence analysis revealed that all strains belonged to the *Betaarterivirus suid1* species, previously known as PRRSV type I, and were likely imported from Europe at least 6 years ago, evolving independently thereafter. When and how the first introduction occurred could not be determined due to the absence of other African sequences for comparison.

**Discussion:**

The present work provides the first detection and characterization of PRRSV molecular epidemiology in Namibia. Based on the present findings, the presence of the PPRSV appears marginal and limited to backyard farms. While biosecurity measures applied in industrial farms appear to be effective in preventing viral introduction, PRRSV circulation in rural settings still represents a potential threat, and considering the socio-economical implication of livestock diseases decreasing animal performances in rural areas, active monitoring should be encouraged to promptly act against emerging menaces and guarantee the welfare of local pig populations.

## 1 Introduction

The swine industry is experiencing significant growth in several African countries, mirroring an increase in internal demand. Approximately 5% of pigs worldwide are raised in Africa, mostly in sub-Saharan region. Pig production in Namibia, although not the largest on the continent, has an important role in local society and economy (1). Large commercial farms are scattered throughout the country and characterised by advanced management and biosecurity measures, especially targeted at the prevention of African Swine Fever (ASF) introduction (2). However, the majority of local pig production is based on smallholder activities. Pig production contributes significantly to poverty alleviation, female and youth employment and guarantees family and community subsistence and welfare in rural and peri-urban settings (3, 4). According to the data provided by the Namibia Agricultural Union (NAU), local pork production in Namibia accounts for between 45 and 50% of consumption, with the deficit being covered by imports from Europe, Germany and Spain being the main sources. Imports from South Africa have been banned due to the foot-and-mouth disease in August 2022. A total of 14,752 pigs were slaughtered in Namibia between January and April 2023. There is an approximate population of 40,000 pigs in Namibia, primarily located in three districts: Mariental, Windhoek, and Tsumeb.

No African country has yet begun to export pork meat, although a reasonable trade of live animals and pig-derived products is known to occur at regional levels.

The impact of diseases on pigs can result in huge economic consequences for farmer livelihoods and income generation both at the household, community, and regional levels. The impact of diseases results in losses of income to the farmers, and possible closure of markets (4).

The presence and wide circulation of several swine pathogens have been documented in Namibia (5–8). Nevertheless, no information is available on one of the most devastating viral diseases affecting the swine industry in high-income countries: Porcine Reproductive and Respiratory Syndrome (PRRS) (9, 10). PRRS is caused by two viral species, *Betaarterivirus suid 1* and *Betaarterivirus suid 2*, belonging to the genus *Betaarterivirus*, family *Arteriviridae*.^1^ These viruses were previously known as porcine reproductive and respiratory syndrome virus 1 (PRRSV-1), or European type, and porcine reproductive and respiratory syndrome virus 2 (PRRSV-2), or American type (11). They are characterized by a single-stranded, positive-sense RNA [ssRNA(+)] genome of approximately 15 kb. About three-quarters of the genome is occupied by open-reading frame (ORF) 1a and ORF1ab, which encode 14 non-structural proteins, while the terminal region consists of eight partially overlapping ORFs (ORF2a, ORF2b, ORF3, ORF4, ORF5, ORF5a, ORF6 and ORF7) coding for the structural proteins (12). Like other RNA viruses, PRRSV shows a high evolutionary and recombination rate causing the continuous emergence of new variants on which genetic drift and selective pressures can act, leading to the observed genetic, phenotypic and biological heterogeneity of circulating strains (13–15). Because of this high variability and rapid evolution, the sequencing of relatively short genomic regions is enough to provide useful information on the molecular epidemiology of these viruses. Among the different ORFs, ORF5 and ORF7 are the most commonly used to reconstruct epidemiological links among pig farms, commercial producers, regions, countries, etc. (16–18).

PRRSV infection is responsible for reproductive disorders in sows, respiratory signs, decreased average daily gain, and mortality in growing/fattening animals although the impact can vary depending on the viral species and strain and the overall host condition and immune status. Costs associated with PPRSV disease management also include antimicrobial costs related to increased susceptibility to secondary infections, control strategies and vaccination (19).

Despite the relevance of this infection, only limited data are available from Africa (20). No information on PPPRSV was available from Namibia prior to this study. Namibia hosts a remarkable biodiversity, including several wild species that can come in contact with infected domestic animals. In addition to domestic pigs, wild boar is the only other host shown to be susceptible to PRRSV infection although the impact of PRRSV in wildlife is generally considered to be limited. However, no information is currently available on the impact of PPRSV on African wild species especially those that have been shown to be susceptible to infections by other swine pathogens (5–7).

To fill this information gap, several samples were collected from both rural and commercial pig populations, and from wild warthogs, to evaluate the presence of the PRRSV in Namibia and to genetically characterize any strains that might be detected.

## 2 Materials and methods

### 2.1 Sample collection and processing

Samples (tonsils or lymph nodes) were collected from 147 healthy butchered pigs (5–6 months of age, approximately 75–100 kg) between March 2018 and May 2023. All of the animals originated from 15 piggeries, comprising 3 industrial facilities and 12 backyard operations. These piggeries were located in six different regions of Namibia: Khomas, Hardap, Kunene, Omaheke, Otjozondjupa, and Erongo. All of the backyard piggeries involved in the study consisted of 30 to 100 animals and observed a medium level of biosecurity. In contrast, the three industrial piggeries maintained herds of approximately 1,400 animals and adhered to a high and strict level of biosecurity.

Additionally, the tonsils of 55 warthogs, living in the area owned by four livestock farms in the Khomas and Otjozondjupa regions, and undergoing periodic hunting campaigns, were collected at slaughter between June 2019 and June 2023, and included in the study.

Sampling collection was conducted by a veterinarian with a specialization in Veterinary Public Health during the post-mortem inspection at the abattoir. All samples were collected in sterile and properly labeled airtight containers. The tissues were removed from the carcasses using sterile disposable scalpels and metal forceps, which were carefully flamed with a portable Bunsen burner for each sample. After collection, all the samples were transported refrigerated to the laboratory (+4°C). The tonsils or lymph nodes were homogenized in 1 mL of sterile phosphate-buffered saline (PBS) using the TissueLyser LT (Qiagen, Germany). Total RNA was extracted from the homogenized samples using the High Pure Viral Nucleic Acid Kit (Hoffman-La Roche, Switzerland) with an elution volume of 100 μL, following the manufacturer’s instructions.

ORF7 from each sample was amplified using a one-step RT-PCR method as described by Oleksiewicz et al. (21). In brief, ORF7 was amplified using the primer pair ORF7F (5′ GCC CCT GCC CAG CAC G 3′) and ORF7R (5′ TCG CCC TAA TTG AAT AGG TGA 3′), resulting in an amplicon of 637 bp (21). The following thermal profile was applied: reverse transcription at 50°C for 30 min, initial denaturation at 94°C for 2 min, followed by 40 cycles of denaturation at 94°C for 15 s, annealing at 55°C for 20 s, and elongation at 68°C for 50 s. This was followed by a final elongation step at 68°C for 10 min.

### 2.2 Sequencing and phylogenetic analysis

Amplicons of positive samples were purified using a Wizard SV Gel and PCR Clean-Up System (Promega) and sequenced commercially by LGC Genomics (Berlin, Germany). The sequences of the positive samples were edited and assembled using the Staden software package version 2.0.0b8. All obtained sequences were submitted to the GenBank database under accession numbers OR604620-OR604626.

ORF7 sequences were preliminary analyzed using BLAST (22) and thereafter a collection of one thousand related sequences was downloaded and aligned to the Namibian ones using MAFFT (23). A maximum-likelihood phylogenetic tree was reconstructed using IQ-TREE (24) selecting the substitution model with the lowest Akaike information criterion (AIC), calculated by JModelTest (25). Ten thousand ultrafast bootstrap replicates were performed to assess clade reliability.

The time passed between the Namibian clade origin and its detection was estimated by performing a serial coalescent analysis using BEAST 1.10 (26), selecting the relaxed lognormal (27) and the Skygrid (28) as molecular clock and viral population size parameter, respectively. For each analysis, an independent run of 100 million generations was performed. Results were analyzed using Tracer 1.7 (29) after the removal of a burn-in of 20% and accepted only if the estimated sample size (ESS) was greater than 200 and the convergence and mixing were adequate. Parameter estimation was summarized in terms of mean and 95% highest posterior density (95HPD). Maximum clade credibility (MCC) trees were constructed and annotated using TreeAnnotator (BEAST package).

## 3 Results

Seven out of 147 pigs (4.76%; Table 1) coming from 3 of the 15 piggeries involved in the study tested positive for PRRSV by RT-PCR. The ORF7 gene was successfully sequenced in all of the positive samples. None of the 55 warthog samples tested positive for PRRSV. Among the 15 piggeries, only three backyard facilities located in the Hardap Region tested positive for PRRSV with a within-herd prevalence ranging from 14.29 to 30%, while none of the industrial piggeries showed evidence of PRRSV.

**Table 1:** Metadata of the samples included in the study.

Species	Samples	Number	Farm ID	Type of farm	Region	Collection date	PRRSV positive	Sequences obtained
Domestic Pig	Lymph node	9	A	Rural	Khomas	March 2018	0	0
Domestic Pig	Lymph node	5	B	Rural	Khomas	May 2018	0	0
Domestic Pig	Lymph node	21	C	Industrial	Otjozondjupa	August 2018	0	0
Domestic Pig	Lymph node	7	D	Rural	Omaheke	October 2018	0	0
Domestic Pig	Tonsils	15	E	Industrial	Hardap	September 2019	0	0
Domestic Pig	Tonsils	15	F	Industrial	Hardap	November 2019	0	0
Domestic Pig	Tonsils	10	G	Rural	Kunene	November 2019	0	0
Domestic Pig	Tonsils	15	H	Rural	Khomas	May 2020	0	0
Domestic Pig	Tonsils	5	I	Rural	Khomas	August 2022	0	0
Domestic Pig	Tonsils	3	L	Rural	Hardap	February 2023	0	0
Domestic Pig	Tonsils	4	M	Rural	Khomas	February 2023	0	0
Domestic Pig	Tonsils	4	N	Rural	Erongo	February 2023	0	0
Domestic Pig	Tonsils	14	O	Rural	Hardap	May 2023	2	2
Domestic Pig	Tonsils	10	P	Rural	Hardap	May 2023	3	3
Domestic Pig	Tonsils	10	Q	Rural	Hardap	May 2023	2	2
Warthog	Tonsils	35	R	Rural	Khomas	June 2019 – June 2023	0	0
Warthog	Tonsils	11	S	Rural	Khomas	September 2019	0	0
Warthog	Tonsils	3	T	Rural	Khomas	June 2022	0	0
Warthog	Tonsils	6	U	Rural	Otjozondjupa	May 2022	0	0

BLAST analysis demonstrated that the sequences were from viruses that belonged to the *Betaarterivirus suid1* species, with a percentage of identity of approximately 95% with the most closely related sequence.

**Figure 1:**
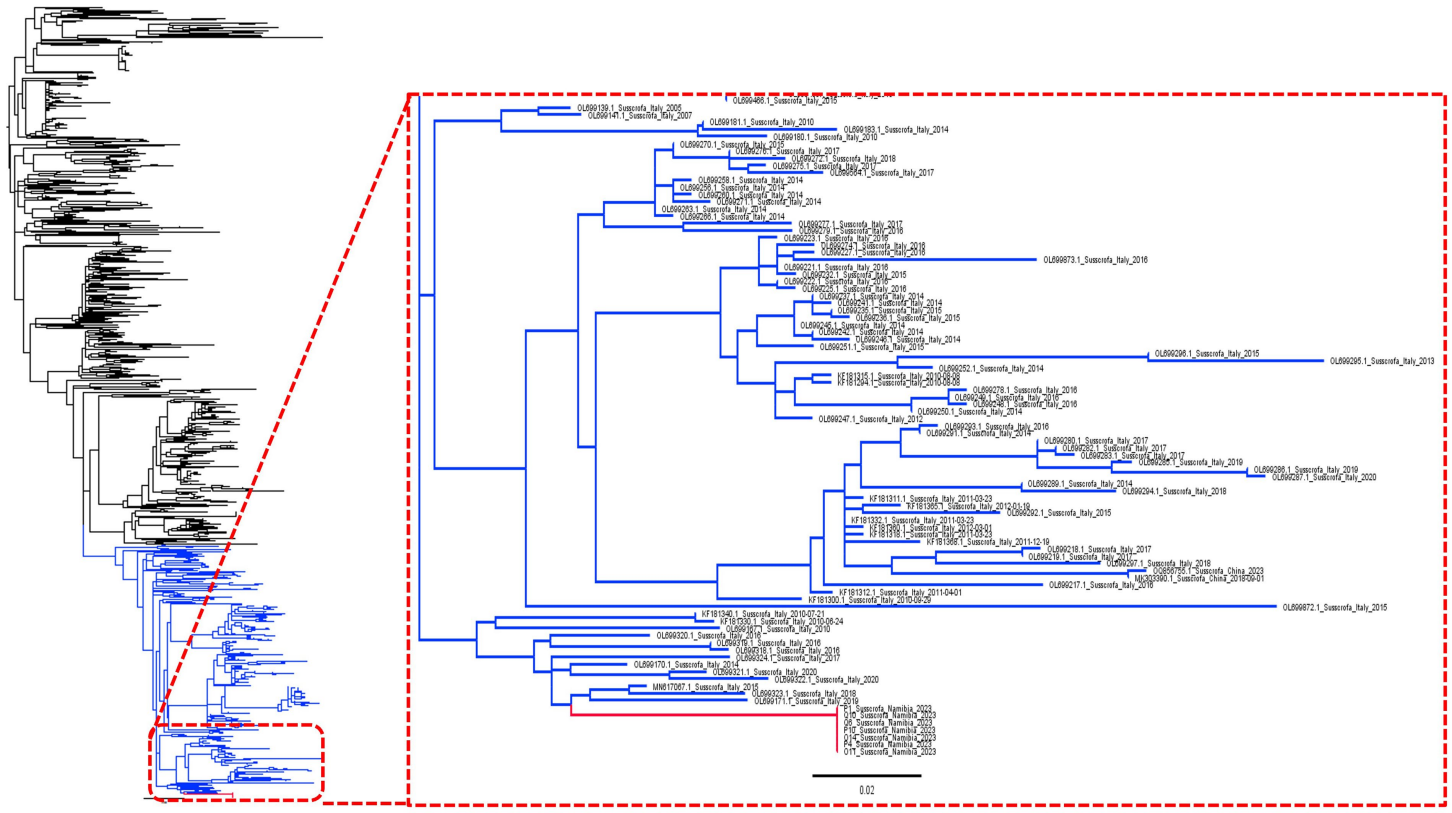
Maximum likelihood phylogenetic tree based on a collection of complete ORF7 sequences. The Namibian clade has been highlighted in red while the clade used for phylodynamic analysis (see Figure 2) is shown in blue.

The phylogenetic analysis confirmed the Namibian strains as being part of a long branch stemming from a European cluster, including viruses collected, in particular, from Italy (Figure 1).

**Figure 2:**
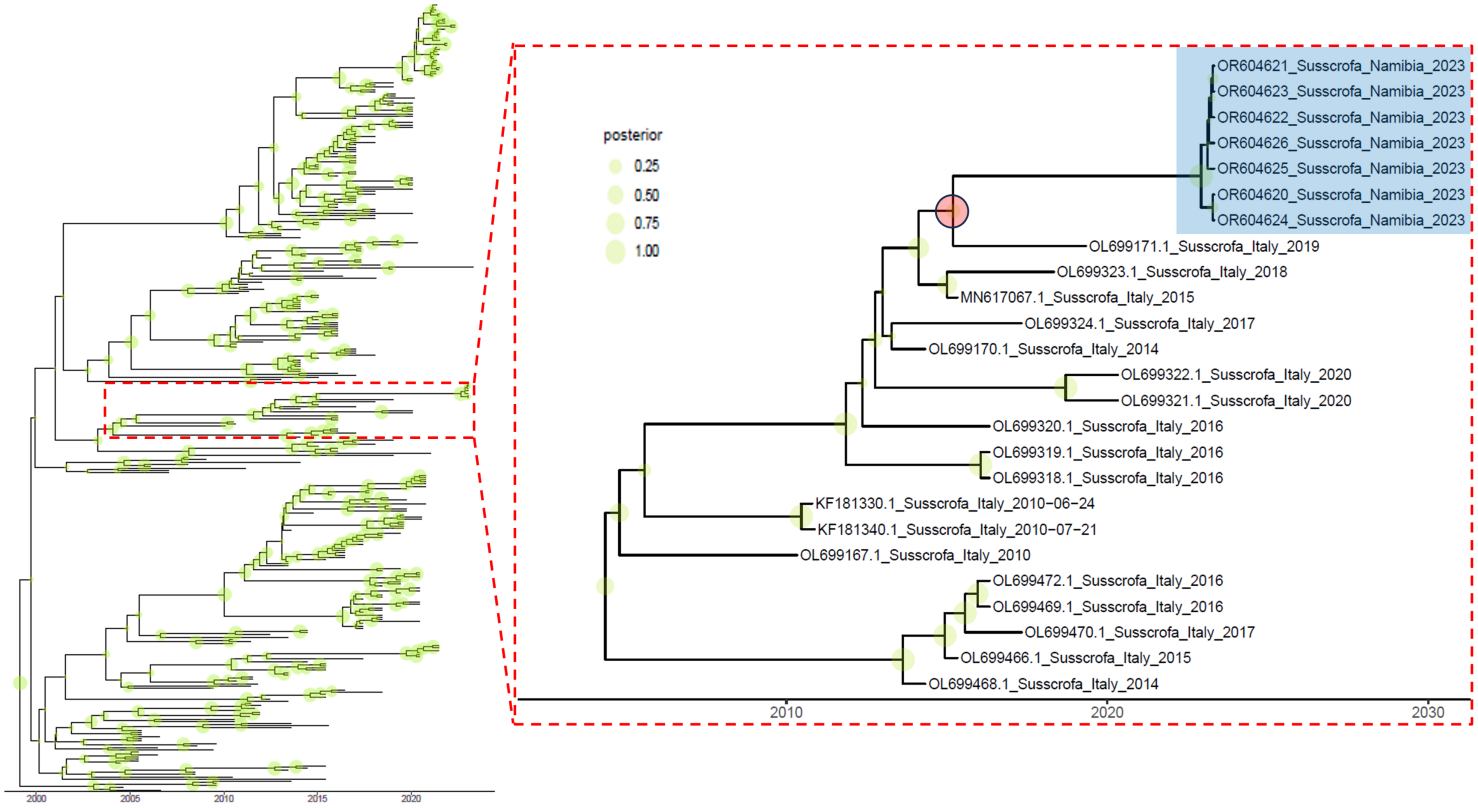
Time-calibrated maximum clade credibility phylogenetic tree based on Namibian and related strains. Shaded green circles whose size is proportional to the posterior probability are reported on the corresponding tree node. The subclade containing the Namibian strains (highlighted in blue) is reported in the right insert. The red circle indicates the likely ancestor and relative divergence time.

Based on the genetic distance and the estimated evolutionary rate (i.e., 6.029∙10^−3^ [95HPD, 5.188∙10^−3^ –6.922∙10^−3^] substitution/site/year), an independent evolution lasting for at least 5.961 years was calculated (mean = 8.011; [95HPD, 5.961–9.743]; Figure 2).

## 4 Discussion

PRRS is one of the most important swine diseases globally, causing an enormous economic burden on the pig industry (19). Although African intensive swine farming only has a marginal impact on the global scenario, both commercial and backyard farms play a significant role in local society, with implications on population welfare and sustenance that goes beyond mere economic relevance (4). High-quality protein source, poverty alleviation, female empowerment are some examples of the multifaceted benefits of pig production in several African countries, Namibia included. Therefore, any disease damaging this sector can have severe detrimental effects on a broader scale. Despite the impact of PPRSV, information on PRRSV epidemiology in Africa is almost absent. The first report of the virus dates back to 2004 from South Africa, followed by a second outbreak in 2007 (20). Some more recent studies have revealed a high detection frequency of PRRSV in Uganda and Nigeria, by serological and molecular methods (30–33). A frequency of PRRSV type 1 and type 2 of 24.65 and 2.73%, respectively, was reported using a commercial real-time RT-PCR assay in Uganda between April 2018 and December 2019 (33).

The present study reports the presence of PRRSV in the Namibian swine population for the first time and, more significantly, the first genetic characterization of African strains.

The overall prevalence was significantly lower than that reported in high-income countries and the previous Ugandan study. None of the tested samples originating from industrial farms were positive, which suggests the effectiveness of biosecurity measures applied to control ASF but also preventing PRRSV introduction (8, 34). This finding confirms what has already been reported for PCV-3 in Namibia. On the other hand, three rural farms were shown to be infected, with a within-farm detection frequency higher than 14%. The identified strains were genetically identical. Because of the high evolutionary rate (i.e., 6.029∙10^−3^ [95HPD: 5.188∙10^–3^ −6.922∙10^−3^] substitution/site/year) of PRRSV estimated herein and in agreement with previous studies (15), this finding suggests an extremely recent introduction. The three farms had strong epidemiological links between them due to the sharing of some boars for breeding purposes, a routine that should thus be strongly discouraged and replaced by the use of certified semen. Therefore, the rapid spread of the identified strain is highly plausible and fits well with the remarkable diffusion potential of PRRSV already demonstrated in intensive farms in high-income countries.

When compared to the international scenario, no closely related sequence could be detected. Based on the estimated PRRSV evolutionary rate at least 6 years of independent evolution can be hypothesized. The closest related sequences were part of an Italian clade of PRRV type I. However, it must be stressed that Italy was largely overrepresented in the ORF7 sequence dataset. Therefore, such findings must be evaluated with caution since no trade of pigs or related products, occurrs between Italy and Namibia. On the other hand, PRRSV circulation among European countries is significant (14). Therefore, an introduction of European strains can be proposed with a higher confidence, as already seen for several other animal pathogens including those of swine. Importation from other continents, Asia in particular, often described for other pathogens (35–38), was not observed but should nevertheless be considered and investigated with more extensive studies.

Namibian importation of semen and live animals from Europe and South Africa for breeding purposes has been forbidden since 2019. Nevertheless, a preceding introduction from Europe fits well with the long, independent evolution estimated for the present clade. Where and how this introduction event occurred cannot be determined due to the absence of any comparable molecular data from other African countries. The report of PRRSV type I in Uganda (33) may suggest that PRRSV was introduced into other African countries that have a more developed swine industry and that it then spread to Namibia through transboundary animal movements. However, a direct introduction in Namibia cannot be excluded either. More intensive sampling and sequencing of PRRSVs in other African countries should be performed to better understand the molecular epidemiology of the virus on the continent, and understand its population dynamics, introduction and spreading patterns.

Only a few farms, located in a restricted geographical area and with strong epidemiological links between each other were shown to be infected, in the absence of detectable clinical signs. This evidence, combined with the PRRSV-free status of intensive farms, suggests a limited circulation of the virus in Namibia. Unfortunately, despite efforts, funding and farmer compliance prevented a more extensive investigation in the current study. Similarly, the inclusion of more Namibian regions would be useful to increase the representativeness of the obtained data. Further studies are therefore needed to gain a more comprehensive understanding of the epidemiological scenario, characterization of risk factors and calculation of actual economic impact.

Finally, no evidence of the presence of PRRSV in warthogs was found, supporting the restricted host tropism of this virus. Alternatively, the apparently low levels of PRRSV circulation in Namibia may also explain why the virus has not been detected in other species.

The present work is the first step in the study and characterization of PRRSV molecular epidemiology in Africa. Although the presence of PRRSV type I strains, most likely originating from Europe, has been shown in Namibia, its relevance seems marginal and limited to backyard farms. While biosecurity measures such as limited access for people and vehicles, the regular cleaning and disinfection of facilities, the maintenance of a closed herd, farm compartmentalization, the presence of fences around the facilities and the daily inspection of animals applied in industrial farms appear effective in preventing viral introduction, PPRSV circulation in rural farms still represents a potential threat to industrial ones, as has been previously shown in high-income countries (17). Moreover, because of the socio-economical implication of livestock diseases decreasing animal performances even in rural areas, active monitoring should be suggested to promptly act against emerging menaces and guarantee local population welfare.

## Data availability statement

The datasets presented in this study can be found in online repositories. The names of the repository/repositories and accession number(s) can be found at: https://www.ncbi.nlm.nih.gov/genbank/, OR604620, OR604621, OR604622, OR604623, OR604624, OR604625, OR604626.

## Ethics statement

The animal studies were approved by Neudamm Decentralized Ethics Committee. The studies were conducted in accordance with the local legislation and institutional requirements. Written informed consent was not obtained from the owners for the participation of their animals in this study because the samples used for the research activity “first detection and molecular characterization of porcine reproductive and respiratory virus (PRRSV) in Namibia, Africa” were secondary samples obtained from domestic pigs and warthogs, which were originally collected during previous research projects conducted in Namibian pig abattoirs and authorized by the Neudamm Decentralized Ethics Committee of the University of Namibia. Therefore, no specific ethical approval for this study was required.

## Author contributions

UM: Conceptualization, Funding acquisition, Investigation, Resources, Supervision, Writing – original draft, Writing – review & editing. LC: Data curation, Formal analysis, Writing – review & editing. MH: Data curation, Writing – review & editing. BC: Data curation, Writing – review & editing. SK: Data curation, Project administration, Writing – review & editing. WD: Funding acquisition, Methodology, Resources, Writing – review & editing. GF: Conceptualization, Data curation, Formal analysis, Methodology, Software, Writing – original draft, Writing – review & editing.

## Footnotes

^1^ https://ictv.global/taxonomy
